# Feasibility of explainable machine learning for analyzing drop landing strategies: effects of landing height and fatigue

**DOI:** 10.3389/fphys.2026.1911268

**Published:** 2026-09-03

**Authors:** Chenglin Liu, Xuan Liu, Siyan Mi, Yujie Liu, Yuan Tian, Youwei Zhang, Xiao Zhang, Lei Pang

**Affiliations:** 1 Institute of Artificial Intelligence in Sports, Capital University of Physical Education and Sports, Beijing, China; 2 Shanghai Research Institute of Sports Science (Shanghai Anti Doping Agency), Shanghai, China; 3 Quartermaster Engineering Technology Research Department, Systems Engineering Institute, Academy of Military Sciences, People’s Liberation Army, Beijing, China

**Keywords:** biomechanical features, discriminant model, drop landing strategy, interpretable machine learning, variable importance

## Abstract

The drop landing (DL) process is a complex neuromuscular control process influenced by multiple factors, among which fatigue state and landing height play significant roles. This study aimed to investigate the influence mechanisms of these two factors on lower-limb landing strategies and to examine the feasibility of explainable machine learning methods in analyzing landing strategies. Joint angles and joint moments of the hip, knee, and ankle in the sagittal plane, as well as vertical ground reaction force (vGRF), were collected under different landing conditions (40 cm and 80 cm heights and their corresponding fatigue states). Cohen’s d effect size analysis was applied to examine group differences in each feature across the time series, and seven classification models were constructed based on multi-dimensional biomechanical features to identify different landing states. Meanwhile, SHapley Additive exPlanations (SHAP) method was employed to analyze feature importance and interactions. Results showed that the Light Gradient Boosting Machine achieved classification accuracies above 0.915 across all four tasks, verifying the feasibility of machine learning methods for landing strategy analysis. Temporal effect size analysis revealed that differences induced by changes in landing height were mainly concentrated in the early landing phase (0%–40%). In contrast, the fatigue effect resulted in a wider distribution and higher magnitude of regions with large effect sizes. SHAP analysis indicated that the knee joint moment had the highest importance across all classification tasks, vGRF contributed prominently in fatigue-related tasks, and feature interaction patterns differed significantly across tasks. In conclusion, explainable machine learning methods can effectively identify and explain differences in DL strategies under various landing conditions, providing a theoretical basis and methodological support for sports injury risk assessment and training strategy optimization.

## 1 Introduction

Drop landing (DL) is a common technical movement in many sports, particularly those requiring explosive power and coordination. During the landing phase, the peak magnitude of ground impact force ranges from three to seven times body weight (Zhang et al., 2019). This movement requires athletes to absorb and disperse the impact forces generated upon landing through coordinated movements of the lower limb joints (hip, knee, and ankle), thereby reducing injury risk (Saadat et al., 2022; Wilzman et al., 2025). However, the effectiveness of landing strategies is influenced by multiple factors, among which fatigue and landing height play critical regulatory roles. Fatigue reduces the excitability of the central nervous system (André et al., 2026), leading to proprioceptive loss (Liu et al., 2023), delayed musculoskeletal responses, and altered lower-extremity biomechanical characteristics (Abbasi et al., 2025). As an important external variable, landing height directly determines the impact load experienced upon landing. Different drop heights result in differences in initial contact velocity, thereby directly affecting landing mechanics and the coordination strategies adopted (Nordin and Dufek, 2017; Howe et al., 2019). Both height and fatigue, individually and synergistically, compel individuals to adjust their landing strategies, potentially shifting from active buffering to a stiff landing pattern, thereby increasing the risk of lower-extremity injury (Dickin et al., 2014).

To elucidate the effects of fatigue and height factors on landing strategies, researchers often employ multidimensional biomechanical indicators, including kinematics, kinetics, and electromyography, for analysis (Xia et al., 2017; Zhang et al., 2019; Zhang et al., 2025). However, findings from existing studies are mostly based on traditional discrete variable analysis, which extracts a few characteristic values, such as peak angles and peak forces, from continuous time series for statistical comparison. Although this processing approach is convenient to apply, it faces a critical bottleneck in that the continuously changing information along the time dimension is largely discarded during the dimensionality reduction process (Gonabadi et al., 2024). When fatigue and height factors act together, it is difficult for single-time-point variables to fully characterize the synergistic response mechanisms of landing strategies. Furthermore, neuromuscular regulation pathways vary across different landing conditions, making it challenging to effectively distinguish between these conditions using only mean values, peak values, or time-specific indicators (Zhang et al., 2024).

Therefore, there is an urgent need for analytical methods that preserve the structural characteristics of time series, to deeply reveal the regulatory mechanisms of fatigue and landing height, both individually and synergistically, on lower-extremity movement strategies. Artificial intelligence algorithms, with their strong high-dimensional data processing and nonlinear mapping capabilities, can directly extract deep features from raw time series to characterize and identify complex movement patterns. In studies focusing on the DL process, multi-layer temporal convolutional neural networks can automatically extract temporal features of kinematic data from inertial measurement unit data, achieving accurate classification of different landing types (Shang et al., 2025). For the estimation of kinematic and kinetic parameters, architectures based on multi-layer perceptrons and long short-term memory neural networks can predict movement trajectories and joint angles of the hip, knee, and ankle, and further back-predict ground reaction forces and joint moments, with prediction accuracy highly consistent with that of three-dimensional motion capture systems and force plates (Chaaban et al., 2021; Asaeda et al., 2024). Nevertheless, the internal decision-making mechanisms of these models are difficult for researchers to understand and interpret, which poses a constraint in application scenarios such as sports biomechanics that demand a high level of mechanistic understanding (Xiang et al., 2025).

Explainable machine learning methods have provided an effective solution to this dilemma. The SHapley Additive exPlanations (SHAP) method, based on the game-theoretic Shapley value, has been applied to interpret injury risk assessment models. Using support vector machines combined with SHAP, researchers have quantified the contribution of various gait biomechanical parameters in the diagnosis of anterior cruciate ligament (ACL) injuries. Core ACL-related features were identified, including maximum hip flexion angle, knee extension moment, and peak ground reaction force (Kokkotis et al., 2022). Using an artificial neural network-based classification model, explainable machine learning methods also identified key features that distinguish landing patterns before and after fatigue. The analysis revealed a compensatory mechanism, namely that fatigue alters lower limb loading patterns, shifting key features from joint angles to joint moments (Xu et al., 2024). The attribution framework and quantitative explanatory power of these methods provide accurate and interpretable decision support for injury prevention, rehabilitation training, and sports performance enhancement.

Although some studies have employed machine learning methods to assist in analyzing strategies in sports or training, existing research on the DL movement pattern has not fully explored the dynamic adjustment mechanisms of landing strategies under the synergistic effects of fatigue and height while preserving the structural characteristics of time series. Moreover, model interpretability remains weak, and there is a lack of precise quantification of the contribution of each biomechanical variable. This study contributes by integrating feature attribution and interaction analyses based on SHAP with a height and fatigue DL design, enabling the examination of how the relative importance and interaction patterns of biomechanical variables vary across different landing demands. The present study mainly carries out the following work: (1) Constructing recognition models of the landing process using various artificial intelligence algorithms based on multi-dimensional time-series features corresponding to four landing conditions involving changes in landing height and fatigue status; (2) Analyzing the distribution of temporal differences in the intensity of each biomechanical feature during the landing phase under different heights and fatigue conditions; (3) Comparing weighted contributions of biomechanical features and their interactions under varying heights and fatigue status, supplying quantitative evidence for the understanding of landing strategy adjustments. The overall framework of this study is illustrated in **Figure 1**.

## 2 Materials and methods

**Figure 1:**
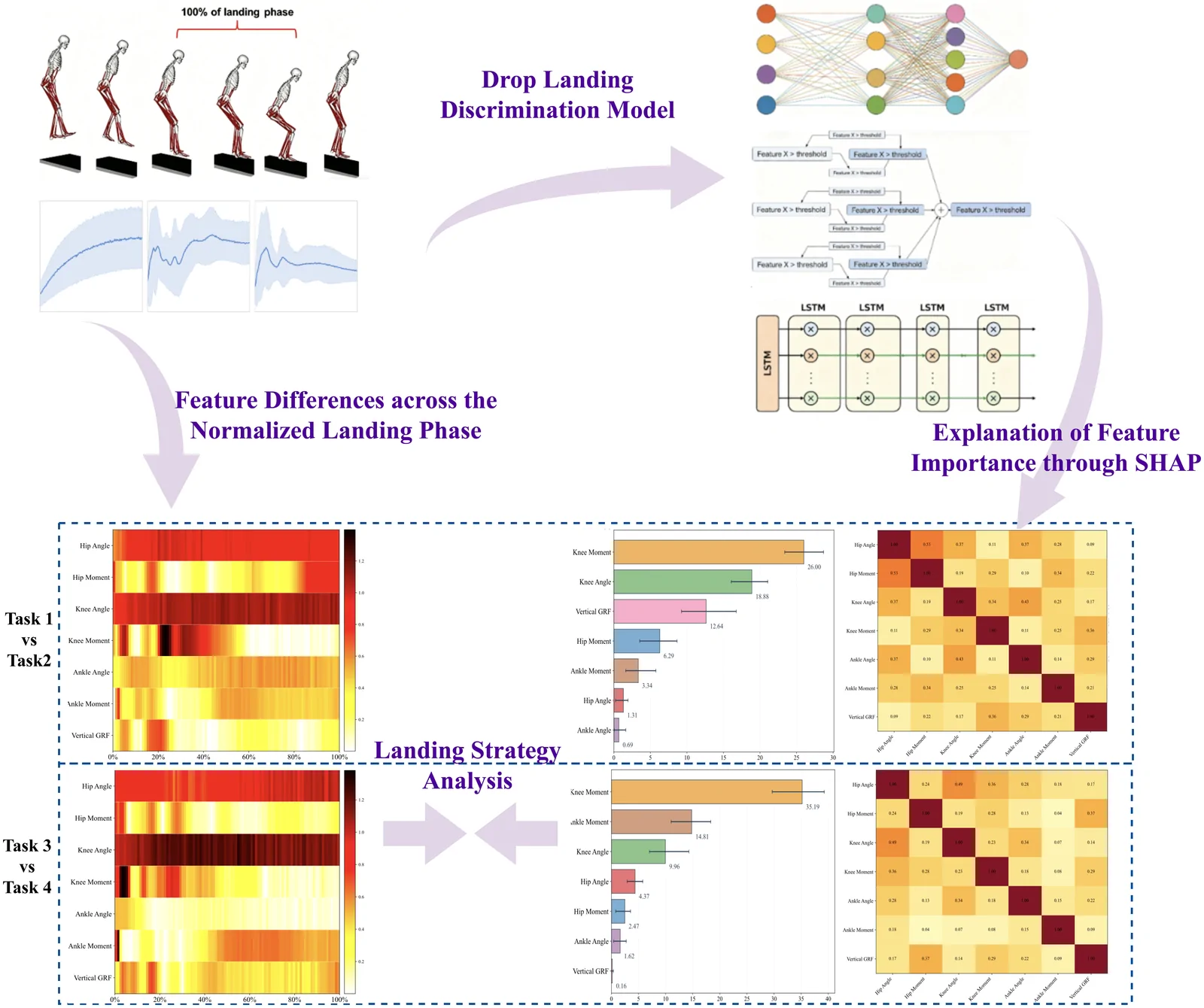
Flow chart of DL control strategy analysis based on interpretable machine learning.

### 2.1 Participants

This study recruited 20 healthy male college students as participants. The inclusion criterion was that participants were non-professional athletes who were able to complete the experimental tasks. The exclusion criteria included lower limb injuries, respiratory system disorders, nervous system diseases, or cardiovascular diseases within the past six months. The study was approved by the Ethics Committee of Capital University of Physical Education and Sports (approval number: 2022A01) and strictly followed its guidelines and regulations. Before the commencement of the study, participants were fully informed of the experimental purpose, procedures, and precautions, particularly the potential risks involved. All participants signed written informed consent forms before their participation, and no strenuous physical activity was performed within 48 hours before testing.

### 2.2 Data collection

Before testing, participants received technical guidance, including instruction and practice of the DL maneuver and the fatigue induction protocol. After completing a 10-minute warm-up, reflective markers were attached to 26 bony landmarks (**Supplementary Table S1**). Before starting the movement, each participant was required to stand still to allow for the acquisition of a static model, which would be used later to generate a musculoskeletal model tailored to the individual’s anatomical characteristics. Participants were instructed to stand on platforms at heights of 40 cm and 80 cm, slowly move their feet to the edge of the platform, and then drop down naturally, with each foot landing on a separate force plate, until the knee reached maximum flexion and stabilized to complete DL. Participants were instructed to keep their arms naturally at their sides and to avoid active arm swinging during landing, in order to standardize upper limb movement across trials. To induce fatigue, participants performed weighted squats at a frequency of 20 repetitions per minute, with a load equal to 30% of their body weight. Each set lasted 1 minute, followed by 1 minute of rest between sets. This process continued until the participant could no longer keep pace with the metronome or complete the movement. Auxiliary criteria for fatigue included heart rate (HR) reaching above 85% of age predicted maximum heart rate (HRmax = 220 − age) or a rating of perceived exertion of 17 or higher on the Borg 6 to 20 Rating of Perceived Exertion (RPE) scale. After fatigue induction, participants repeated the tests at drop heights of 40 cm and 80 cm. During post fatigue testing, heart rate and RPE were reassessed before each drop height condition to confirm the fatigue state. Each of the four landing conditions was repeated three times to ensure data stability. During the experiment, data were collected using the following equipment: two three-dimensional force plates (Kistler, Winterthur, Switzerland), sampling at 1000 Hz to obtain ground reaction forces in three directions, and an eight-camera high-speed infrared motion capture system (OptiTrack, Natural Point, USA) with a sampling frequency of 200 Hz to capture three-dimensional movement trajectories. In summary, four types of DL conditions were collected: pre-fatigue 40 cm (Task 1), pre-fatigue 80 cm (Task 2), post-fatigue 40 cm (Task 3), and post-fatigue 80 cm (Task 4). A 2×2 matrix analysis was then performed, consisting of Task 1 vs. Task 2, Task 3 vs. Task 4, Task 1 vs. Task 3, and Task 2 vs. Task 4, to compare the effects of height and fatigue on landing strategy.

### 2.3 Data processing

A fourth-order Butterworth low-pass filter was applied to smooth the kinematic trajectories and force plate signals, with cutoff frequencies set to 10 Hz and 50 Hz, respectively. The time series of the DL phase from initial ground contact was normalized to 0–100% (at intervals of 0.5%) representing the movement cycle, where 0% corresponded to the instant of ground contact and 100% corresponded to the moment of maximum knee flexion during DL. Ground reaction forces (GRF) and joint moments were normalized by body mass (BM). Based on this processing, four data matrices of size 60 × 1407 were constructed as inputs for the machine learning models. Here, 60 represents the 3 repetitions for each of the 20 participants, and 1407 represents the data from seven features, namely hip angle, hip moment, knee angle, knee moment, ankle angle, ankle moment, and vertical ground reaction force (vGRF), across 201 consecutive time points during the landing process.

### 2.4 Identification model and model evaluation

Logistic regression is a classic binary classification statistical model that maps the linear combination of independent variables to the probability of event occurrence through the Sigmoid function, with output values strictly ranging between 0 and 1. The model parameters are estimated using maximum likelihood estimation, and it does not require the independent variables to follow a normal distribution, nor does it impose assumptions on the distribution of the data (Couronne et al., 2018). In this study, the regularization parameter C of the logistic regression is set to 1.

A support vector machine (SVM) is a supervised learning model whose core idea is to map the input space to a high-dimensional feature space via a kernel function and find an optimal hyperplane that maximizes the margin between classes (Huang et al., 2021). This hyperplane is defined by the decision function given in Equation 1:

(1) $$f(x)=w^{T}\varphi (x)+b$$

where, *w* is the weight vector, *b* is the bias, and *ϕ*(*x*) represents the feature map implicitly defined by the kernel function. By introducing the kernel trick, SVM can effectively map low-dimensional linearly inseparable data into a high-dimensional space where it becomes linearly separable (Sheykhmousa et al., 2020). Therefore, it demonstrates unique advantages in handling small sample sizes, nonlinearity, and high-dimensional pattern recognition problems. In this study, the kernel function used is the RBF kernel, and the penalty parameter C is set to 1.0.

A multilayer perceptron (MLP) is a classic feedforward artificial neural network composed of an input layer, one or more hidden layers, and an output layer, with neurons between layers being fully connected by weights (Mondal et al., 2024). In each layer, neurons perform a weighted sum of the output from the previous layer and then introduce a nonlinear transformation through a nonlinear activation function. In this study, the MLP uses ReLU as the activation function and Adam as the optimizer. It has two hidden layers with 100 and 50 neurons respectively, and a learning rate of 0.001, with these hyperparameters being determined through grid search.

Random forest (RF) is an ensemble learning method based on decision trees. It generates multiple sample subsets from the original training set using bootstrap sampling and introduces random feature selection at each node split to reduce model variance (Song, 2015). At each node split, RF first randomly selects a subset of features from all features and then chooses the optimal split feature from this subset (Wang, 2023), thereby reducing the correlation between trees. In this study, the number of treesis set to 150. To prevent overfitting, at least two samples are required to continue splitting a node.

Light Gradient Boosting Machine (LightGBM) is a gradient boosting decision tree framework, with its core innovations including a histogram-based algorithm and a leaf-wise growth strategy with depth constraints (Sheng et al., 2024; Zhang et al., 2025). Unlike traditional level-wise growth, LightGBM selects the node with the largest split gain from all current leaves at each step to perform splitting, thereby achieving lower error under the same number of iterations. The objective function can be expressed by Equation 2:

(2) $$L^{\left(t\right)}=\sum_{i=1}^{n}l(y_{i},{\hat{y}}_{i}^{\left(t-1\right)}+f_{t}(x_{i}))+\Omega (f_{t})$$

where *f_t_* is the decision tree added at the *t*-th round, and Ω is the regularization term. In this paper, the core parameters of this model were determined through grid search and set as follows: the maximum number of leaves per tree is 15, the learning rate is 0.01, and the number of boosting rounds is 200.

A long short term memory network (LSTM) is a special type of recurrent neural network (RNN). It is specifically designed to address the vanishing gradient and exploding gradient problems that traditional RNNs face when processing long sequence data (Chekhmane and Benali, 2025). At each time step, the LSTM unit receives the current input and the hidden state from the previous time step, and performs computations through three gate structures: the input gate controls the inflow of new information, the forget gate determines which old information to discard, and the output gate regulates the output from the memory cell to the hidden state. In this study, the LSTM network has two layers, with the number of neurons in each layer set to 64 and 32, respectively. Tanh and Adam are used as the activation function and optimizer, respectively. A dropout rate of 0.3 is applied to prevent overfitting, and batch normalization is implemented to avoid gradient explosion. The learning rate is set to 0.001, the batch size to 8, and the maximum number of epochs to 150, with each epoch representing one complete pass through the training samples in the corresponding training fold. Finally, landing state classification is achieved through softmax.

**Figure 2:**
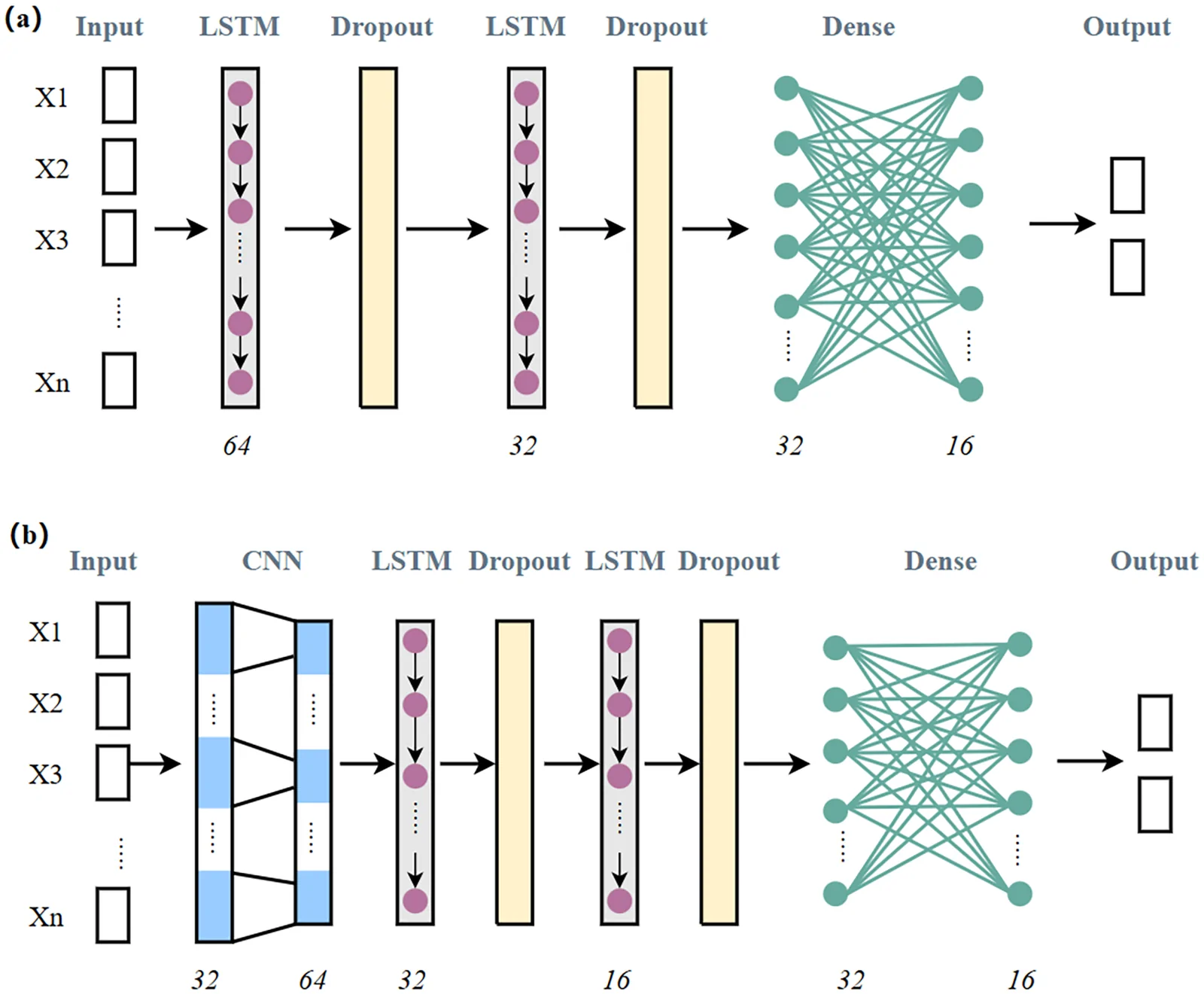
Network architecture diagram of deep learning algorithms: **(A)** LSTM; **(B)** 1DCNN-LSTM.

A convolutional neural network (CNN) is a type of feedforward neural network specialized for processing grid-structured data. Its design is inspired by the biological visual cortex mechanism (Jin et al., 2025). The core characteristic of CNN is the use of convolution operations to extract local features. The convolutional layer slides multiple convolutional kernels over the input data and performs convolution operations to extract different features. The pooling layer aggregates the feature maps output by the convolutional layer. In this study, max pooling is adopted to achieve feature dimensionality reduction and spatial invariance. A 1DCNN-LSTM model is used in this study, which can simultaneously characterize the deep features and temporal features of kinematic and kinetic parameters (Hu et al., 2026). Both the CNN and LSTM have two layers, with the number of neurons set to 32, 64, 32, and 16 respectively. The convolutional kernel size is 1×3, the convolution stride is 1, and the activation function is tanh. The learning rate is set to 0.001, the batch size to 8, and the maximum number of epochs to 150. Considering the relatively small sample size and the comparative purpose of the analysis, hyperparameters were predefined using commonly adopted baseline or default settings. This strategy was intended to reduce task specific overfitting and maintain comparability across classification tasks. The network architectures of the LSTM and 1DCNN-LSTM models are presented in **Figure 2**. For the LSTM and 1DCNN-LSTM models, the original 60×1407 matrix was reshaped into a three dimensional tensor with the format samples×time steps×features (60×201×7).

To construct and evaluate the model, participants were randomly divided into training and testing sets at a ratio of 4:1. A five-fold cross-validation strategy was subsequently employed, wherein in each iteration, four folds were used for model training and the remaining fold served as the testing set. The final performance of the model was determined by averaging the results obtained from all five folds. To prevent data leakage, the data partitioning was performed in a participant-wise manner, ensuring that data from the same subject were not concurrently assigned to both the training and testing sets.

### 2.5 Interpretable model

SHapley Additive exPlanations (SHAP) is an explainable machine learning method based on cooperative game theory. Its core idea is to fairly distribute the model’s prediction outcome to each input feature, assigning each feature a numerical value that quantifies its contribution—the SHAP value. This method is built upon the Shapley value and calculates the influence of each feature on a specific prediction by considering the feature’s marginal contribution across all possible combinations (Salih et al., 2025). From a mathematical perspective, for a model *f* containing *M* features, the SHAP value *ϕ*(*i*) of the *i-*th feature is computed by Equation 3:

(3) $$\phi_{i}(f,x)=\sum_{S\subseteq \left\{1,\ldots ,M\right\}\backslash \left\{i\right\}}\frac{\left|S\right|!(M-\left|S\right|-1)!}{M!}\left[f_{x}(S\cup \left\{i\right\})-f_{x}(S)\right]$$

where *S* is a subset of features that does not contain the *i-*th feature, and *f_x_*(*S*) denotes the expected prediction of the model for sample *x* given the feature subset *S*. This formula computes the weighted average of the change in model prediction when feature *i* is added to all possible feature combinations, with the weight determined by the number of permutations of the combination.

In global feature importance analysis, SHAP measures the overall impact of each feature on the model output by calculating its mean absolute SHAP value across all samples (Khajavian et al., 2026). The global feature importance *I_j_* is defined by Equation 4:

(4) $$I_{j}=\frac{1}{N}\sum_{i=1}^{N}\left|\phi_{j}^{\left(k\right)}\right|$$

in which, *N* is the total number of samples, and *ϕ_j_*(*k*) represents the SHAP value of the *j*-th feature on the k-th sample. This metric reflects the global contribution strength of a feature to the prediction outcome, with larger values indicating greater feature importance. Compared with traditional permutation importance or Gini importance, SHAP can more fairly handle correlations between features, avoiding underestimation of a feature’s true contribution due to information redundancy (Lundberg et al., 2020).

In addition, SHAP can reveal the relationship between individual feature values and their corresponding SHAP values to uncover feature interactions. For features *i* and *j*, their interaction SHAP value *ϕ_i_*,*_j_* can be defined as the additional contribution generated when both features appear together. The calculation formula is given in Equation 5:

(5) $$\phi_{i,j}=\sum_{S\subseteq \left\{1,\ldots ,M\right\}\backslash \left\{i\right\}}\frac{\left|S\right|!(M-\left|S\right|-2)!}{(M-1)!}\left[f_{x}(S\cup \left\{i,j\right\})-f_{x}(S\cup \left\{i\right\})+f_{x}(S)\right]$$

By introducing a second feature through color encoding, one can intuitively observe how the two features jointly affect the prediction outcome, thereby revealing synergistic or inhibitory relationships between variables (Grinsztajn et al., 2022).

### 2.6 Software

In this paper, OpenSim 4.2 was used to construct a musculoskeletal model for calculating joint angles and joint moments during movement. The implementation and training of the identification model and interpretable model were performed using Python 3.7 in the PyCharm software.

## 3 Results

### 3.1 Kinematic and dynamic characteristic changes during drop landing

**Figure 3:**
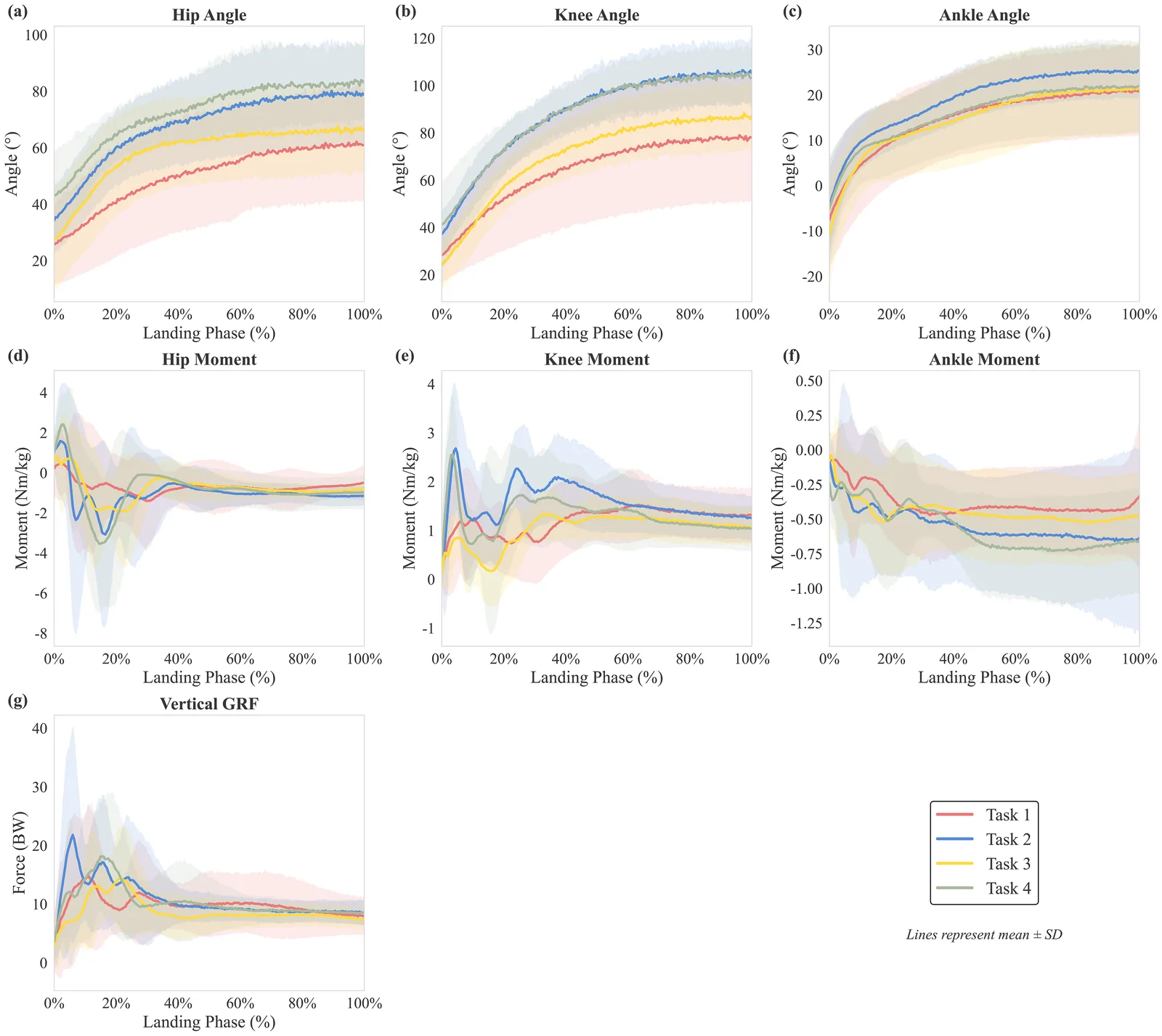
Changes in kinematic and kinetic characteristics during DL: **(A–C)** Sagittal plane joint angles; **(D–F)** Sagittal plane joint moments; **(G)** vGRF.

The basic characteristics of the 20 participants were: age, 20.05 ± 1.49 years; height, 175.68 ± 5.63 cm; and body mass, 74.18 ± 12.29 kg. This study first analyzed the changes in sagittal plane hip, knee, and ankle joint angles, joint moments, and vGRF during DL. Joint moments and vGRF were normalized to body mass, and the results are presented in **Figure 3**. As the landing phase progressed, the angles of all three lower limb joints exhibited an increasing trend. During the time-normalized landing phase, the hip and knee joint angles showed clear condition-dependent differences, with Task 4 generally exhibiting the largest differences, followed by Task 2. The ankle joint angle also varied across tasks, but its pattern was less distinct than those of the hip and knee joints. Among the kinematic variables, the hip joint angle showed the visual separation between tasks. Unlike joint angles, the joint moments of the three lower limb joints and the vGRF displayed trend differences across conditions, yet their curves overlapped considerably, making intuitive differentiation difficult. Specifically, the ankle joint moment and vGRF were difficult to distinguish during the 40%–100% interval of the landing phase, while the ankle joint moment alone was difficult to distinguish during the 0%–40% interval. Given the considerable data overlap described above, relying on intuitive data changes alone is insufficient to effectively distinguish the influences of fatigue and landing height on landing strategy. Consequently, models featuring greater quantitative capability and interpretability are necessary to gain deeper insights into the mechanisms of strategic adjustment.

### 3.2 Model construction for landing pattern recognition

**Figure 4:**
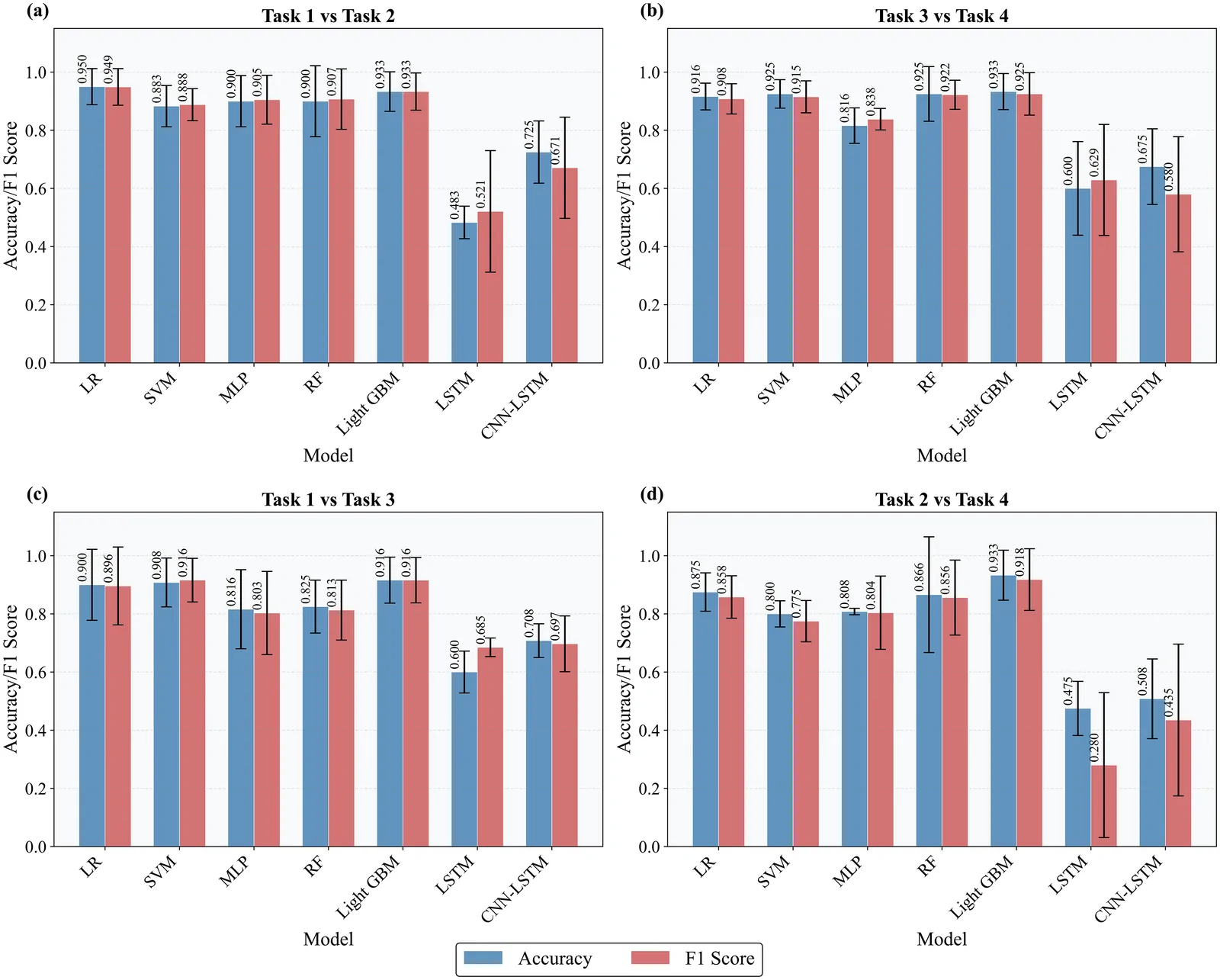
Comparison of classification performance across different models: **(A)** Task 1 vs. Task 2; **(B)** Task 3 vs. Task 4; **(C)** Task 1 vs. Task 3; **(D)** Task 2 vs. Task 4.

Based on multidimensional kinematic and kinetic features, multiple algorithms were employed to construct recognition models of the landing process under different landing conditions. Comparisons were made among classical machine learning methods (LR, SVM, and MLP), ensemble learning algorithms (RF and LightGBM), and deep learning algorithms (LSTM and CNN-LSTM). The modeling results are presented in **Figure 4**, including accuracy (ACC) and F1 score (F1).

Overall, ACC and F1 varied across models and tasks. Ensemble learning algorithms generally achieved higher average performance in most tasks, with LightGBM obtaining the highest or near-highest mean ACC and F1 values across multiple tasks; RF also demonstrated relatively stable performance in tasks involving the discrimination of height. Among traditional machine learning algorithms, LR, SVM and MLP showed task-dependent performance variations. Specifically, LR achieved the highest classification performance in Task 1 vs. Task 2 and maintained relatively good results in Task 3 vs. Task 4; however, model performance declined in tasks involving fatigue. SVM attained ACC and F1 scores comparable to those of LightGBM in Task 1 vs. Task 3, while MLP also yielded relatively high average performance in Task 1 vs. Task 2. In contrast, CNN-LSTM and LSTM showed lower overall classification performance, with more pronounced variability across tasks.

In Task 1 vs. Task 2, RF achieved the highest ACC and F1, while LightGBM, RF, MLP, and SVM also maintained relatively high ACC and F1 values, indicating that multiple models could effectively distinguish between the two task conditions. In Task 3 vs. Task 4, the ACC and values of F1 LightGBM, RF, LR, and SVM were all exceeding 0.9. For Task 1 vs. Task 3, LightGBM and SVM obtained higher ACC and F1 values than the other models, followed by LR. In Task 2 vs. Task 4, LightGBM achieved the highest mean ACC and F1, followed by LR and RF. In contrast, CNN-LSTM and LSTM showed lower ACC and F1 values than the other models.

In addition, Friedman omnibus tests and Holm-corrected pairwise Wilcoxon comparisons were performed to evaluate performance differences among the above models, with detailed statistical results provided in the **Supplementary Materials**. The omnibus tests indicated overall differences among models across tasks and evaluation metrics. However, after Holm correction, no pairwise comparisons between models reached statistical significance (**Supplementary Tables S2**, **S3**).

To further validate the proportion of misclassified samples per task, the predictive performance of the LightGBM model, which demonstrated relatively balanced performance, was evaluated on the test set (**Tables 1**, **2**). For the height variation in Task 1 vs. Task 2, the model demonstrated strong discriminative ability, achieving recognition accuracies of 93.3% and 93.2% for Task 1 and Task 2, respectively. Regarding Task 3 vs. Task 4, the model achieved accuracies of 96.7% and 90.0%, respectively, with 3.3% of Task 3 samples misclassified as Task 4 and 10.0% of Task 4 samples misclassified as Task 3. For the fatigue-related comparison in Task 1 vs. Task 3, the model achieved recognition accuracies of 91.4% and 91.7%, respectively, indicating relatively stable discrimination between the non-fatigued and fatigued states under this landing condition. As for Task 2 vs. Task 4, the model achieved a recognition accuracy of 100.0% for Task 2, while 13.3% of Task 4 samples were misclassified as Task 2. Overall, the difficulty of correctly identifying different landing conditions varied across tasks, with the greatest confusion observed in the Task 2 vs. Task 4 comparison.

**Table 1:** Classification prediction results of LightGBM under different height factors.

Predicted label True label	Task 1 vs. Task 2		Predicted label True label	Task 3 vs. Task 4	
	Task 1	Task 2		Task 3	Task 4
Task 1	93.3%	6.7%	Task 3	96.7%	3.3%
Task 2	6.8%	93.2%	Task 4	10.0%	90.0%

**Table 2:** Classification prediction results of LightGBM under fatigue factors.

Predicted label True label	Task 1 vs. Task 3		Predicted label True label	Task 2 vs. Task 4	
	Task 1	Task 3		Task 2	Task 4
Task 1	91.4%	8.6%	Task 2	100.0%	0.0%
Task 3	8.3%	91.7%	Task 4	13.3%	86.7%

### 3.3 Biomechanical feature differences across the normalized landing phase

**Figure 5:**
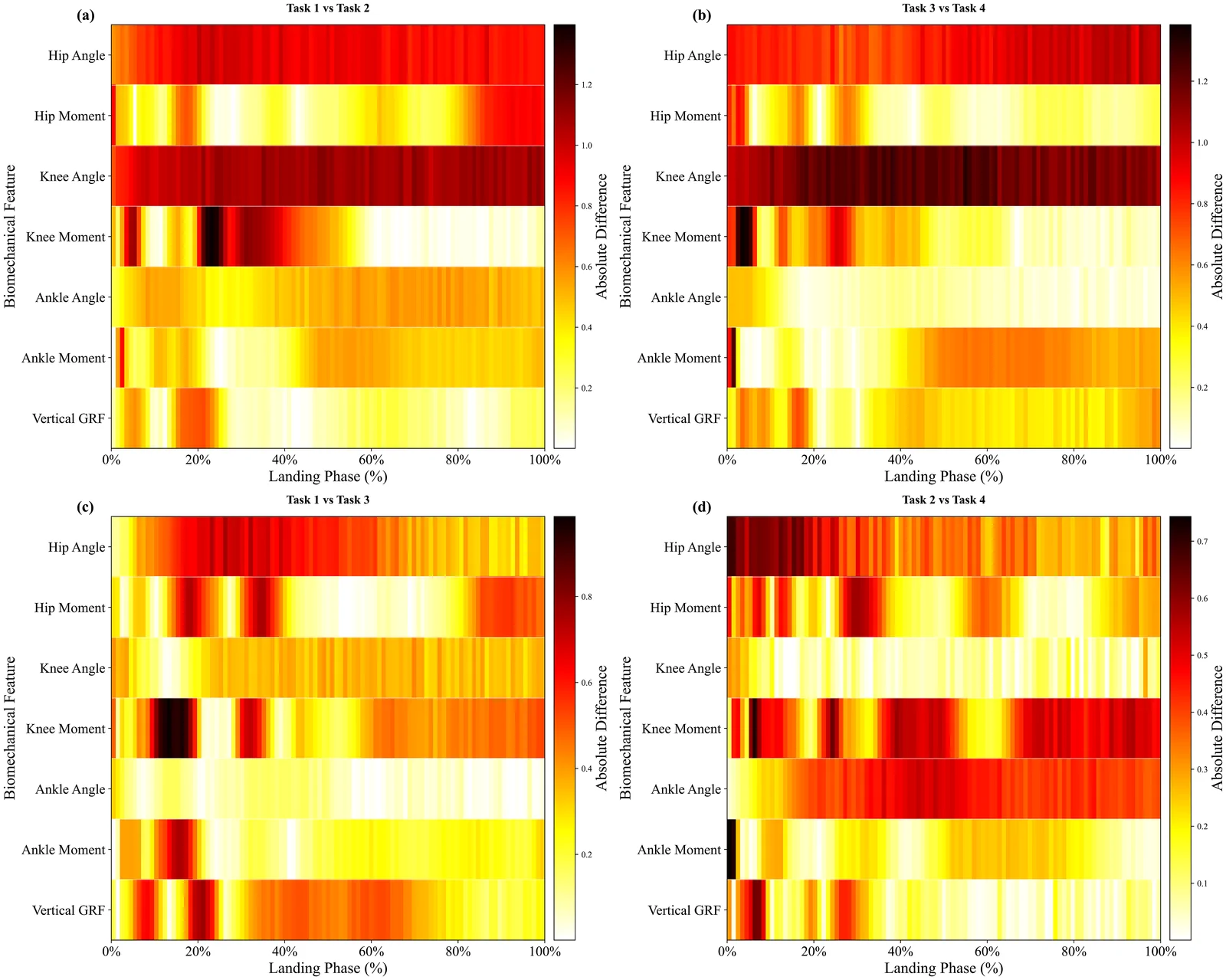
Effect size distribution of biomechanical feature differences across the normalized DL phase: **(A)** Task 1 vs. Task 2; **(B)** Task 3 vs. Task 4; **(C)** Task 1 vs. Task 3; **(D)** Task 2 vs. Task 4.

The results from Section 3.2 indicate that landing height and fatigue influenced landing strategies, with kinematic and kinetic features showing both similarities and differences across conditions. To further examine these condition-related differences, point-by-point Cohen’s d was calculated across the time-normalized DL phase to characterize the effect size distribution of biomechanical differences at equivalent relative phases of landing. As shown in **Figure 5a**, for Task 1 vs. Task 2, the differences were mainly concentrated in the 0%–40% portion of the normalized landing phase, where hip angle, knee angle, and knee moment showed relatively large effect-size regions. Toward the later normalized phase, the magnitude of these differences gradually decreased. Compared with Task 1 vs. Task 2, Task 3 vs. Task 4 showed a broader distribution and greater magnitude of high-difference regions, particularly in hip angle, knee angle, hip moment, and knee moment. This suggests that the combined influence of fatigue and landing height produced more pronounced biomechanical differences across the normalized landing phase. Under fixed-height comparisons, the fatigue-related difference pattern was different. For Task 1 vs. Task 3, the effect-size distribution was more dispersed and was not limited to the early normalized landing phase. Hip moment and knee moment showed relatively large differences across much of the normalized phase. For Task 2 vs. Task 4, the overall magnitude of differences was the most prominent, with large effect-size regions observed across nearly the entire first 40% of the normalized landing phase. Hip angle, ankle moment, vGRF, and knee moment showed particularly strong differences between the two tasks.

### 3.4 Analysis of overall importance of features based on SHAP

**Figure 6:**
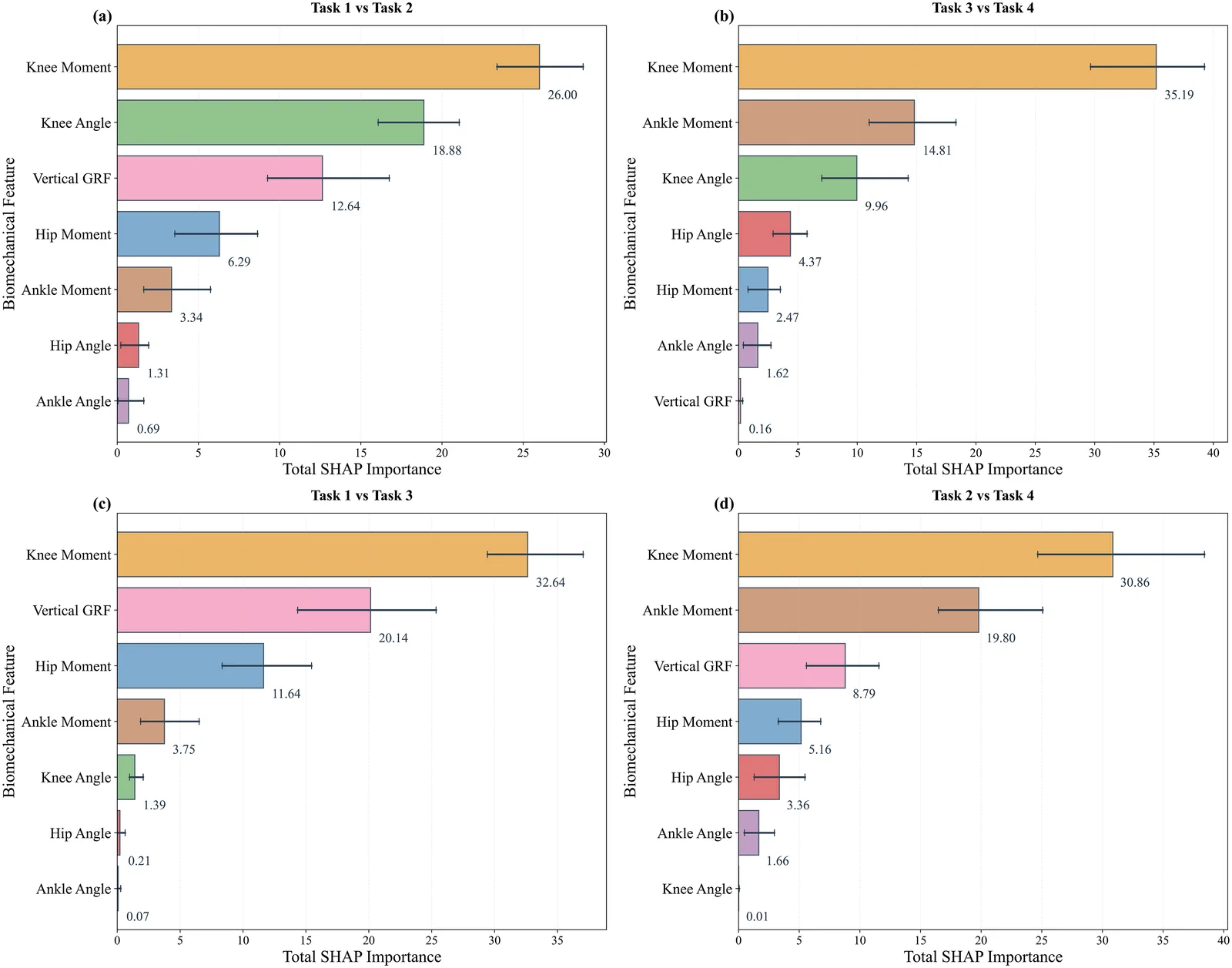
SHAP-based feature importance analysis of the DL process under different conditions: **(A)** Task 1 vs. Task 2; **(B)** Task 3 vs. Task 4; **(C)** Task 1 vs. Task 3; **(D)** Task 2 vs. Task 4.

Although biomechanical features differed across the four landing conditions, partial overlap among these features may have contributed to misclassification in the landing-state recognition models. To enhance the robustness of the interpretability results, SHAP values were computed using a LightGBM classifier that demonstrated relatively high and stable performance, and the final feature importance ranking was determined by averaging the feature contribution estimates across the five cross-validation folds. **Figure 6** presents the SHAP importance values and their corresponding standard deviations for each biomechanical feature (LR-based feature importance shown in **Supplementary Figure S1**). The SHAP-based feature importance analysis across the four classification tasks revealed clear differences in the dominant biomechanical features. For the Task 1 vs. Task 2, which distinguishes landing height, knee moment and knee angle, made the most prominent contributions. In the Task 3 vs. Task 4, which distinguishes height under fatigued conditions, knee moment played a dominant role. For the Task 1 vs. Task 3, which distinguishes fatigue state at the same landing height, both knee moment and vGRF contributed significantly. In the Task 2 vs. Task 4, knee moment, ankle moment, and vGRF were the most important features. Overall, knee moment consistently showed high importance across all four classification tasks. The vGRF made significant contributions to tasks involving fatigue-state discrimination, especially Task 1 vs. Task 3 and Task 2 vs. Task 4. Ankle moment also contributed substantially in Task 3 vs. Task 4 and Task 2 vs. Task 4, whereas hip-related features generally played auxiliary roles.

**Figure 7:**
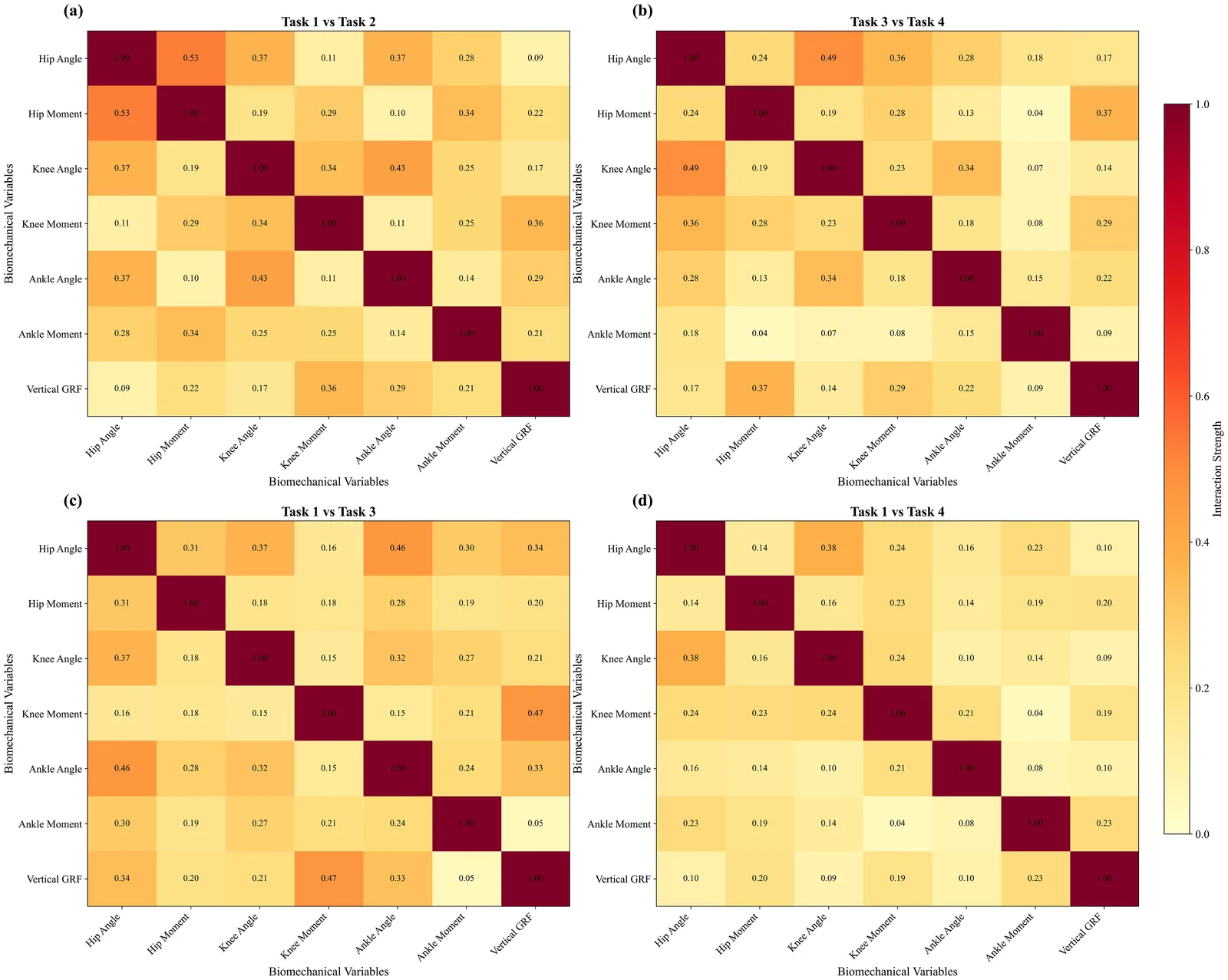
Heatmap of biomechanical variable interaction matrix: **(A)** Task 1 vs. Task 2; **(B)** Task 3 vs. Task 4; **(C)** Task 1 vs. Task 3; **(D)** Task 2 vs. Task 4.

The pairwise interaction patterns among the seven biomechanical features were examined across the four landing-task comparisons using heatmaps, as shown in **Figure 7**. Overall, the interaction strengths varied across tasks and were mostly within the low-to-moderate range. For Task 1 vs. Task 2, feature interactions were generally weak to moderate, with relatively higher interactions observed between knee angle and ankle angle, hip angle and knee angle, and knee moment and vertical GRF. For Task 3 vs. Task 4, interactions involving hip and knee features became more evident, particularly the interaction between hip angle and knee angle. Moderate interactions were also observed for selected pairs, such as hip angle with knee moment and knee angle with ankle angle, indicating that the interaction pattern was not limited to a single joint feature. For Task 1 vs. Task 3, the interaction pattern was more concentrated around knee loading and vertical GRF, with the knee moment-vGRF pair showing the most prominent interaction in this comparison. In contrast, Task 2 vs. Task 4 showed weaker and more dispersed interactions, without a clearly dominant high-strength interaction involving vGRF. Overall, these results suggest task-dependent interaction patterns among biomechanical features, with the knee moment-vGRF interaction playing an important role in fatigue-related landing-state discrimination.

## 4 Discussion

This study compared classical machine learning, ensemble learning, and deep learning models to assess their performance in classifying landing states. By combining Cohen’s d effect size heatmaps with the SHAP method, the study further uncovered the spatiotemporal evolution of biomechanical features across different landing conditions and their respective contributions to model classification.

### 4.1 Complexity of landing strategies reflected by model performance differences

This study found that LightGBM and RF achieved the best overall performance in landing state classification tasks based on multidimensional time-series features, with high stability and classification accuracy across different conditions. In contrast, LSTM and 1DCNN-LSTM did not demonstrate the expected advantages in time-series modeling. This finding is largely consistent with recent studies on structured biomechanical data. It has been reported that under conditions of moderate or even relatively small sample sizes, tree-based models often maintain an advantage over deep learning models in handling nonlinear relationships, feature interactions, and complex decision boundaries (Grinsztajn et al., 2022; Liew et al., 2024). In this study, LightGBM achieved relatively high and balanced accuracy across multiple classification tasks. Considering the limited sample size and relatively high feature dimensionality of the DL biomechanical dataset, these findings suggest that LightGBM may be a suitable modeling option for the current data setting.

This result is partially contrary to the common view that deep learning models are naturally suitable for time-series data processing (Ordóñez and Roggen, 2016; Fawaz et al., 2019). However, this discrepancy does not imply that the landing process lacks exploitable temporal dependencies. A more reasonable explanation should be derived from the data characteristics of the landing task itself. Drop landing is essentially a short-duration impact task, in which GRF and joint angles undergo abrupt changes at the instant of ground contact, exhibiting typical features of strong non-stationarity, instantaneous mutations, and peak dominance. LSTM models excel at capturing slow, smooth, or medium-to-long-term trend dependencies. In contrast, for such impact-dominant signals that heavily rely on precise peak alignment, these models require a large number of accurately labeled samples and sophisticated temporal alignment strategies to learn effectively (Dindorf et al., 2024). Under the practical constraints of limited sample size and minor misalignments in ground contact timing across individuals, LSTM struggles to extract stable and reliable temporal patterns and is prone to overfitting or underfitting. In comparison, tree-based ensemble models do not rely on strict point-by-point temporal alignment. Instead, they capture key information during the impact phase through window-based statistical features, exhibiting greater robustness to signal mutations. Therefore, the suboptimal performance of LSTM and CNN-LSTM essentially reflects a mismatch among task characteristics, data conditions, and the preferences of temporal models.

From this perspective, the findings of this study support the view that, in biomechanical classification tasks with limited sample sizes and dominated by short-duration impacts, ensemble learning methods tend to be more reliable and robust than deep temporal models. Model selection should not be based solely on theoretical structural advantages, but rather on a comprehensive consideration of data characteristics, such as task duration, degree of abruptness, alignment precision, and sample size. This conclusion provides a practical methodological reference for future research on DL and the analysis of similar impact-based movements.

### 4.2 Intrinsic relationship between task difficulty and feature importance

To explore the differences in task difficulty across classification tasks and their underlying biomechanical causes, the recognition performance of LightGBM on landing patterns across different tasks was compared. Distinguishing between different landing heights was relatively easy, with recognition accuracies of 93.3% for Task 1 and 93.2% for Task 2 in the Task 1 vs. Task 2 comparison, and 96.7% for Task 3 and 90.0% for Task 4 in the Task 3 vs. Task 4 comparison. In contrast, the identification of fatigue states showed more task-dependent performance rather than uniformly lower accuracy. In the Task 1 vs. Task 3 comparison, the model achieved comparable accuracies for both conditions, whereas in the Task 2 vs. Task 4 comparison, 13.3% of Task 4 samples were misclassified as Task 2, suggesting greater confusion under this specific condition.

Regarding the effect of landing height, the present findings are largely consistent with previous studies. It has been shown that as landing height increases, variables such as ground reaction force, knee flexion angle, angular velocity, and joint power undergo systematic changes (Maniar et al., 2022; Bi et al., 2024). In other words, height variation itself introduces relatively clear changes in external mechanical load, and the body tends to adopt relatively consistent movement adjustments in response to such load changes. Consequently, the models in this study distinguished changes in landing height more easily. By contrast, the results under fatigued conditions were more task-dependent. In the latter two classification tasks, substantial overlap was observed between the two conditions, resulting in higher misclassification rates. These findings are consistent with recent systematic reviews and experimental studies. It has been reported that the effects of fatigue on landing biomechanics do not always change in a uniform direction; different fatigue induction protocols, landing tasks, and participant characteristics may lead to divergent outcomes (Wong et al., 2020; Abbasi et al., 2025; He et al., 2025). For example, some studies reported increased peak vGRF after fatigue, while others found decreased peak vGRF under specific protocols (Wong et al., 2020; Bi et al., 2024; He et al., 2025; Zheng et al., 2025). Therefore, the occasional difficulty in identifying fatigue states in this study does not imply that the effects of fatigue are negligible. Rather, it may suggest that fatigue-induced biomechanical changes are more variable and less stable across landing conditions.

### 4.3 Neuromuscular synergistic adaptation mechanisms revealed by feature interactions

According to the SHAP results, knee moment consistently showed high discriminative importance across the pairwise classification tasks. In comparisons involving different landing heights, knee-related features played a particularly important role. Specifically, knee moment and knee angle were the dominant features in Task 1 vs. Task 2, whereas knee moment remained dominant in Task 3 vs. Task 4, accompanied by an increased contribution from ankle moment. These findings are consistent with the established role of the knee joint in landing control (Wong et al., 2020; He et al., 2025), while also suggesting that the discriminative information used by the model may shift under fatigued conditions.

In fatigue-state discrimination tasks, the importance of vGRF became more evident, particularly in Task 1 vs. Task 3 and Task 2 vs. Task 4. However, this effect was not uniform across all task comparisons involving fatigue, as vGRF contributed minimally to Task 3 vs. Task 4. This indicates that vGRF may be more informative for distinguishing fatigue state than for distinguishing landing height under fatigue. In Task 2 vs. Task 4, knee moment, ankle moment, and vGRF constituted the main discriminative features, suggesting that fatigue-related classification may rely on information related to both joint loading and impact force characteristics. This interpretation is broadly consistent with previous evidence showing that fatigue can alter landing biomechanics and affect lower-limb joint loading and impact-related variables (Abbasi et al., 2025). Nevertheless, SHAP importance reflects model-based feature reliance and should not be directly equated with physiological mechanisms.

The feature interaction results further showed that the role of vGRF was task-dependent. In Task 1 vs. Task 3, the interaction between knee moment and vGRF was among the most pronounced, suggesting that the model relied on their combined information when distinguishing fatigue state at the same landing height. In contrast, vGRF-related interactions were weaker in Task 2 vs. Task 4, despite the relatively high SHAP importance of vGRF. This dissociation suggests that, in this comparison, vGRF may contribute more through its main effect rather than through strong interactions with joint-level features. Overall, the interaction patterns support a task-dependent interpretation of biomechanical feature use, rather than a fixed fatigue-induced coordination pattern, which is also consistent with the heterogeneous fatigue effects reported in previous landing studies (Abbasi et al., 2025; He et al., 2025; Sarvestan and Rad, 2025; Zheng et al., 2025).

### 4.4 Strengths and limitations

This study has these key strengths. First, it integrated full time-series biomechanical information with explainable machine learning, which reduced the information loss associated with relying only on discrete variables. Second, the use of SHAP analysis provided interpretable information regarding the biomechanical features contributing to landing state classification.

Nevertheless, this study has several limitations. First, the participant sample was relatively homogeneous, which may limit the generalizability of the findings. Second, the biomechanical indicators were limited by the absence of multiplanar kinematic and electromyographic data. Third, the loaded squat protocol used in this study was designed to induce general whole body fatigue rather than fatigue specific to landing tasks. Therefore, the fatigue related findings should be interpreted as reflecting model discrimination under a general fatigued state and may not fully represent biomechanical adaptations to landing specific fatigue. Finally, SHAP analysis identifies the information used by the model for classification, but it does not establish causal mechanisms. In addition, the SHAP analysis in this study was based on a relatively well-performing LightGBM classifier. Future studies should compare SHAP-based feature importance patterns across multiple classifiers to assess the robustness of model interpretability. Larger and more diverse samples, broader biomechanical indicators, and task-specific fatigue protocols are also needed to further validate the practical applicability of this approach in sports training and injury risk monitoring.

## 5 Conclusion

This study examined drop landing strategies under different landing height and fatigue conditions using full time-series biomechanical features and explainable machine learning. The results supported the feasibility of this approach for characterizing differences in landing strategies and showed that LightGBM provided effective classification performance across task comparisons. Compared with traditional discrete-variable analysis, integrating full time-series information with explainable machine learning may better preserve dynamic biomechanical patterns during landing. Knee joint moment was consistently identified as an important discriminative feature, while vGRF demonstrated greater importance in fatigue-state discrimination. Knee angle and ankle moment also contributed to specific task comparisons, suggesting that landing-state recognition depends on both joint-level mechanics and impact-related information. Overall, these findings indicate that explainable machine learning based on continuous biomechanical features can provide useful insights into landing strategy adaptations under different task conditions. This approach may help clarify the relationships among key biomechanical features, training-related adaptations, and injury risk, thereby facilitating the translation of biomechanical findings into sports training and rehabilitation practice.

## Data Availability

The raw data supporting the conclusions of this article will be made available by the authors, without undue reservation.
