# Supplementary material for: Feasibility of explainable machine learning for analyzing drop landing strategies: effects of landing height and fatigue
Source: Front Physiol. 2026 Sep 3;17:1911268. doi: 10.3389/fphys.2026.1911268 (PMC13581516; doi:10.3389/fphys.2026.1911268)
Supplement: Supplementary file 1 [file Supplementaryfile1.docx]

Table S1. Name and location of landmark points

| Landmark Name | Location |
| --- | --- |
| V. Cervical (C7) | Posterior neck |
| V. Sacral | L4-L5 spinous process |
| L.Acromium / R. Acromium | Left acromion / Right acromion |
| L. ASIS / R. ASIS | Anterior superior iliac spine |
| L.Thigh.Upper /R. Thigh.Upper | Lateral thigh |
| L. Thigh.Rear / R. Thigh.Rear | Anterior thigh |
| L.Knee.Lat / R.Knee.Lat | Lateral border of the patella |
| L. Knee.Med / R. Knee.Med | Medial border of the patella |
| L. Shank.Lat / R. Shank.Lat | Lateral border of the tibia |
| L. Ankle.Lat / R. Ankle.Lat | Lateral malleolus |
| L. Ankle.Med / R. Ankle.Med | Medial malleolus |
| L. Toe.Tip / R. Toe.Tip | Midpoint between the second and third toes |
| L. Toe.Lat / R. Toe.Lat | Fifth metatarsal |
| L. Heel / R. Heel | Calcaneal tuberosity |

Table S2. Friedman's overall test for classification tasks

| Task | Metric | N_Models | Statistic | P_Value | Kendall_W |
| --- | --- | --- | --- | --- | --- |
| Task 1 vs. Task 2 | ACC | 7 | 15.209581 | 0.009503 | 0.608383 |
| Task 1 vs. Task 2 | F1 | 7 | 15.449102 | 0.008606 | 0.617964 |
| Task 3 vs. Task 4 | ACC | 7 | 20.542169 | 0.000988 | 0.821687 |
| Task 3 vs. Task 4 | F1 | 7 | 19.704142 | 0.001420 | 0.788166 |
| Task 1 vs. Task 3 | ACC | 7 | 16.676471 | 0.005156 | 0.667059 |
| Task 1 vs. Task 3 | F1 | 7 | 14.797688 | 0.011263 | 0.591908 |
| Task 2 vs. Task 4 | ACC | 7 | 20.952381 | 0.000827 | 0.838095 |
| Task 2 vs. Task 4 | F1 | 7 | 20.518293 | 0.000999 | 0.820732 |

Note: N_Models = number of compared models; Statistic = Friedman test statistic; P_Value = p-value of the Friedman test; Kendall_W = Kendall’s coefficient of concordance.

Table S3. Pairwise Wilcoxon comparison of six models

| Task | Metric | Model A | Model B | P_Uncorrected | P_Holm |
| --- | --- | --- | --- | --- | --- |
| Task 1 vs. Task 2 | ACC | RL | CNN-LSTM | 0.125000 | 0.435238 |
| Task 1 vs. Task 2 | ACC | RL | LSTM | 0.062500 | 0.375000 |
| Task 1 vs. Task 2 | ACC | RL | LightGBM | 0.179712 | 0.435238 |
| Task 1 vs. Task 2 | ACC | RL | RF | 0.285049 | 0.435238 |
| Task 1 vs. Task 2 | ACC | RL | MLP | 0.108809 | 0.435238 |
| Task 1 vs. Task 2 | ACC | RL | SVM | 0.067889 | 0.375000 |
| Task 1 vs. Task 2 | ACC | CNN-LSTM | LSTM | 0.062500 | 0.937500 |
| Task 1 vs. Task 2 | ACC | CNN-LSTM | LightGBM | 0.125000 | 1 |
| Task 1 vs. Task 2 | ACC | CNN-LSTM | RF | 0.125000 | 1 |
| Task 1 vs. Task 2 | ACC | CNN-LSTM | MLP | 0.125000 | 1 |
| Task 1 vs. Task 2 | ACC | CNN-LSTM | SVM | 0.125000 | 1 |
| Task 1 vs. Task 2 | ACC | LSTM | LightGBM | 0.062500 | 0.937500 |
| Task 1 vs. Task 2 | ACC | LSTM | RF | 0.062500 | 0.937500 |
| Task 1 vs. Task 2 | ACC | LSTM | MLP | 0.062500 | 0.937500 |
| Task 1 vs. Task 2 | ACC | LSTM | SVM | 0.062500 | 0.937500 |
| Task 1 vs. Task 2 | ACC | LightGBM | RF | 0.592980 | 1 |
| Task 1 vs. Task 2 | ACC | LightGBM | MLP | 0.285049 | 1 |
| Task 1 vs. Task 2 | ACC | LightGBM | SVM | 0.065600 | 0.937500 |
| Task 1 vs. Task 2 | ACC | RF | MLP | 1 | 1 |
| Task 1 vs. Task 2 | ACC | RF | SVM | 0.580712 | 1 |
| Task 1 vs. Task 2 | ACC | MLP | SVM | 0.812500 | 1 |
| Task 1 vs. Task 2 | F1 | RL | CNN-LSTM | 0.125000 | 0.435238 |
| Task 1 vs. Task 2 | F1 | RL | LSTM | 0.062500 | 0.375000 |
| Task 1 vs. Task 2 | F1 | RL | LightGBM | 0.179712 | 0.435238 |
| Task 1 vs. Task 2 | F1 | RL | RF | 0.285049 | 0.435238 |
| Task 1 vs. Task 2 | F1 | RL | MLP | 0.108809 | 0.435238 |
| Task 1 vs. Task 2 | F1 | RL | SVM | 0.067889 | 0.375000 |
| Task 1 vs. Task 2 | F1 | CNN-LSTM | LSTM | 0.312500 | 1 |
| Task 1 vs. Task 2 | F1 | CNN-LSTM | LightGBM | 0.125000 | 1 |
| Task 1 vs. Task 2 | F1 | CNN-LSTM | RF | 0.062500 | 0.937500 |
| Task 1 vs. Task 2 | F1 | CNN-LSTM | MLP | 0.125000 | 1 |
| Task 1 vs. Task 2 | F1 | CNN-LSTM | SVM | 0.125000 | 1 |
| Task 1 vs. Task 2 | F1 | LSTM | LightGBM | 0.062500 | 0.937500 |
| Task 1 vs. Task 2 | F1 | LSTM | RF | 0.062500 | 0.937500 |
| Task 1 vs. Task 2 | F1 | LSTM | MLP | 0.062500 | 0.937500 |
| Task 1 vs. Task 2 | F1 | LSTM | SVM | 0.062500 | 0.937500 |
| Task 1 vs. Task 2 | F1 | LightGBM | RF | 0.592980 | 1 |
| Task 1 vs. Task 2 | F1 | LightGBM | MLP | 0.285049 | 1 |
| Task 1 vs. Task 2 | F1 | LightGBM | SVM | 0.067889 | 0.937500 |
| Task 1 vs. Task 2 | F1 | RF | MLP | 1 | 1 |
| Task 1 vs. Task 2 | F1 | RF | SVM | 0.715001 | 1 |
| Task 1 vs. Task 2 | F1 | MLP | SVM | 0.625000 | 1 |
| Task 3 vs. Task 4 | ACC | RL | CNN-LSTM | 0.062500 | 0.375000 |
| Task 3 vs. Task 4 | ACC | RL | LSTM | 0.062500 | 0.375000 |
| Task 3 vs. Task 4 | ACC | RL | LightGBM | 0.592980 | 1 |
| Task 3 vs. Task 4 | ACC | RL | RF | 0.715001 | 1 |
| Task 3 vs. Task 4 | ACC | RL | MLP | 0.108809 | 0.435238 |
| Task 3 vs. Task 4 | ACC | RL | SVM | 0.785495 | 1 |
| Task 3 vs. Task 4 | ACC | CNN-LSTM | LSTM | 0.812500 | 1 |
| Task 3 vs. Task 4 | ACC | CNN-LSTM | LightGBM | 0.062500 | 0.937500 |
| Task 3 vs. Task 4 | ACC | CNN-LSTM | RF | 0.062500 | 0.937500 |
| Task 3 vs. Task 4 | ACC | CNN-LSTM | MLP | 0.062500 | 0.937500 |
| Task 3 vs. Task 4 | ACC | CNN-LSTM | SVM | 0.062500 | 0.937500 |
| Task 3 vs. Task 4 | ACC | LSTM | LightGBM | 0.062500 | 0.937500 |
| Task 3 vs. Task 4 | ACC | LSTM | RF | 0.062500 | 0.937500 |
| Task 3 vs. Task 4 | ACC | LSTM | MLP | 0.125000 | 0.937500 |
| Task 3 vs. Task 4 | ACC | LSTM | SVM | 0.062500 | 0.937500 |
| Task 3 vs. Task 4 | ACC | LightGBM | RF | 0.465209 | 1 |
| Task 3 vs. Task 4 | ACC | LightGBM | MLP | 0.062500 | 0.937500 |
| Task 3 vs. Task 4 | ACC | LightGBM | SVM | 0.715001 | 1 |
| Task 3 vs. Task 4 | ACC | RF | MLP | 0.067889 | 0.937500 |
| Task 3 vs. Task 4 | ACC | RF | SVM | 1 | 1 |
| Task 3 vs. Task 4 | ACC | MLP | SVM | 0.065600 | 0.937500 |
| Task 3 vs. Task 4 | F1 | RL | CNN-LSTM | 0.062500 | 0.375000 |
| Task 3 vs. Task 4 | F1 | RL | LSTM | 0.062500 | 0.375000 |
| Task 3 vs. Task 4 | F1 | RL | LightGBM | 0.592980 | 1 |
| Task 3 vs. Task 4 | F1 | RL | RF | 1 | 1 |
| Task 3 vs. Task 4 | F1 | RL | MLP | 0.144127 | 0.576508 |
| Task 3 vs. Task 4 | F1 | RL | SVM | 0.785495 | 1 |
| Task 3 vs. Task 4 | F1 | CNN-LSTM | LSTM | 1 | 1 |
| Task 3 vs. Task 4 | F1 | CNN-LSTM | LightGBM | 0.062500 | 0.937500 |
| Task 3 vs. Task 4 | F1 | CNN-LSTM | RF | 0.062500 | 0.937500 |
| Task 3 vs. Task 4 | F1 | CNN-LSTM | MLP | 0.062500 | 0.937500 |
| Task 3 vs. Task 4 | F1 | CNN-LSTM | SVM | 0.062500 | 0.937500 |
| Task 3 vs. Task 4 | F1 | LSTM | LightGBM | 0.062500 | 0.937500 |
| Task 3 vs. Task 4 | F1 | LSTM | RF | 0.062500 | 0.937500 |
| Task 3 vs. Task 4 | F1 | LSTM | MLP | 0.125000 | 0.937500 |
| Task 3 vs. Task 4 | F1 | LSTM | SVM | 0.062500 | 0.937500 |
| Task 3 vs. Task 4 | F1 | LightGBM | RF | 1 | 1 |
| Task 3 vs. Task 4 | F1 | LightGBM | MLP | 0.125000 | 0.937500 |
| Task 3 vs. Task 4 | F1 | LightGBM | SVM | 0.715001 | 1 |
| Task 3 vs. Task 4 | F1 | RF | MLP | 0.062500 | 0.937500 |
| Task 3 vs. Task 4 | F1 | RF | SVM | 0.785495 | 1 |
| Task 3 vs. Task 4 | F1 | MLP | SVM | 0.067889 | 0.937500 |
| Task 1 vs. Task 3 | ACC | RL | CNN-LSTM | 0.125000 | 0.750000 |
| Task 1 vs. Task 3 | ACC | RL | LSTM | 0.125000 | 0.750000 |
| Task 1 vs. Task 3 | ACC | RL | LightGBM | 0.785495 | 0.930418 |
| Task 1 vs. Task 3 | ACC | RL | RF | 0.179712 | 0.750000 |
| Task 1 vs. Task 3 | ACC | RL | MLP | 0.144127 | 0.750000 |
| Task 1 vs. Task 3 | ACC | RL | SVM | 0.465209 | 0.930418 |
| Task 1 vs. Task 3 | ACC | CNN-LSTM | LSTM | 0.062500 | 0.937500 |
| Task 1 vs. Task 3 | ACC | CNN-LSTM | LightGBM | 0.062500 | 0.937500 |
| Task 1 vs. Task 3 | ACC | CNN-LSTM | RF | 0.312500 | 1 |
| Task 1 vs. Task 3 | ACC | CNN-LSTM | MLP | 0.144127 | 1 |
| Task 1 vs. Task 3 | ACC | CNN-LSTM | SVM | 0.062500 | 0.937500 |
| Task 1 vs. Task 3 | ACC | LSTM | LightGBM | 0.062500 | 0.937500 |
| Task 1 vs. Task 3 | ACC | LSTM | RF | 0.125000 | 1 |
| Task 1 vs. Task 3 | ACC | LSTM | MLP | 0.067889 | 0.937500 |
| Task 1 vs. Task 3 | ACC | LSTM | SVM | 0.062500 | 0.937500 |
| Task 1 vs. Task 3 | ACC | LightGBM | RF | 0.065600 | 0.937500 |
| Task 1 vs. Task 3 | ACC | LightGBM | MLP | 0.312500 | 1 |
| Task 1 vs. Task 3 | ACC | LightGBM | SVM | 1 | 1 |
| Task 1 vs. Task 3 | ACC | RF | MLP | 1 | 1 |
| Task 1 vs. Task 3 | ACC | RF | SVM | 0.125000 | 1 |
| Task 1 vs. Task 3 | ACC | MLP | SVM | 0.312500 | 1 |
| Task 1 vs. Task 3 | F1 | RL | CNN-LSTM | 0.125000 | 0.652857 |
| Task 1 vs. Task 3 | F1 | RL | LSTM | 0.125000 | 0.652857 |
| Task 1 vs. Task 3 | F1 | RL | LightGBM | 0.592980 | 1 |
| Task 1 vs. Task 3 | F1 | RL | RF | 0.108809 | 0.652857 |
| Task 1 vs. Task 3 | F1 | RL | MLP | 0.273322 | 0.819965 |
| Task 1 vs. Task 3 | F1 | RL | SVM | 0.853923 | 1 |
| Task 1 vs. Task 3 | F1 | CNN-LSTM | LSTM | 0.625000 | 1 |
| Task 1 vs. Task 3 | F1 | CNN-LSTM | LightGBM | 0.062500 | 0.937500 |
| Task 1 vs. Task 3 | F1 | CNN-LSTM | RF | 0.437500 | 1 |
| Task 1 vs. Task 3 | F1 | CNN-LSTM | MLP | 0.125000 | 1 |
| Task 1 vs. Task 3 | F1 | CNN-LSTM | SVM | 0.062500 | 0.937500 |
| Task 1 vs. Task 3 | F1 | LSTM | LightGBM | 0.062500 | 0.937500 |
| Task 1 vs. Task 3 | F1 | LSTM | RF | 0.125000 | 1 |
| Task 1 vs. Task 3 | F1 | LSTM | MLP | 0.187500 | 1 |
| Task 1 vs. Task 3 | F1 | LSTM | SVM | 0.062500 | 0.937500 |
| Task 1 vs. Task 3 | F1 | LightGBM | RF | 0.067889 | 0.937500 |
| Task 1 vs. Task 3 | F1 | LightGBM | MLP | 0.312500 | 1 |
| Task 1 vs. Task 3 | F1 | LightGBM | SVM | 0.715001 | 1 |
| Task 1 vs. Task 3 | F1 | RF | MLP | 0.812500 | 1 |
| Task 1 vs. Task 3 | F1 | RF | SVM | 0.125000 | 1 |
| Task 1 vs. Task 3 | F1 | MLP | SVM | 0.187500 | 1 |
| Task 2 vs. Task 4 | ACC | RL | CNN-LSTM | 0.062500 | 0.375000 |
| Task 2 vs. Task 4 | ACC | RL | LSTM | 0.062500 | 0.375000 |
| Task 2 vs. Task 4 | ACC | RL | LightGBM | 0.144127 | 0.435238 |
| Task 2 vs. Task 4 | ACC | RL | RF | 0.715001 | 0.715001 |
| Task 2 vs. Task 4 | ACC | RL | MLP | 0.108809 | 0.435238 |
| Task 2 vs. Task 4 | ACC | RL | SVM | 0.140773 | 0.435238 |
| Task 2 vs. Task 4 | ACC | CNN-LSTM | LSTM | 1 | 1 |
| Task 2 vs. Task 4 | ACC | CNN-LSTM | LightGBM | 0.062500 | 0.937500 |
| Task 2 vs. Task 4 | ACC | CNN-LSTM | RF | 0.062500 | 0.937500 |
| Task 2 vs. Task 4 | ACC | CNN-LSTM | MLP | 0.062500 | 0.937500 |
| Task 2 vs. Task 4 | ACC | CNN-LSTM | SVM | 0.062500 | 0.937500 |
| Task 2 vs. Task 4 | ACC | LSTM | LightGBM | 0.062500 | 0.937500 |
| Task 2 vs. Task 4 | ACC | LSTM | RF | 0.062500 | 0.937500 |
| Task 2 vs. Task 4 | ACC | LSTM | MLP | 0.062500 | 0.937500 |
| Task 2 vs. Task 4 | ACC | LSTM | SVM | 0.062500 | 0.937500 |
| Task 2 vs. Task 4 | ACC | LightGBM | RF | 0.285049 | 1 |
| Task 2 vs. Task 4 | ACC | LightGBM | MLP | 0.067889 | 0.937500 |
| Task 2 vs. Task 4 | ACC | LightGBM | SVM | 0.067889 | 0.937500 |
| Task 2 vs. Task 4 | ACC | RF | MLP | 0.108809 | 0.937500 |
| Task 2 vs. Task 4 | ACC | RF | SVM | 0.437500 | 1 |
| Task 2 vs. Task 4 | ACC | MLP | SVM | 1 | 1 |
| Task 2 vs. Task 4 | F1 | RL | CNN-LSTM | 0.062500 | 0.375000 |
| Task 2 vs. Task 4 | F1 | RL | LSTM | 0.062500 | 0.375000 |
| Task 2 vs. Task 4 | F1 | RL | LightGBM | 0.273322 | 1 |
| Task 2 vs. Task 4 | F1 | RL | RF | 0.715001 | 1 |
| Task 2 vs. Task 4 | F1 | RL | MLP | 0.273322 | 1 |
| Task 2 vs. Task 4 | F1 | RL | SVM | 0.312500 | 1 |
| Task 2 vs. Task 4 | F1 | CNN-LSTM | LSTM | 0.273322 | 1 |
| Task 2 vs. Task 4 | F1 | CNN-LSTM | LightGBM | 0.062500 | 0.937500 |
| Task 2 vs. Task 4 | F1 | CNN-LSTM | RF | 0.062500 | 0.937500 |
| Task 2 vs. Task 4 | F1 | CNN-LSTM | MLP | 0.062500 | 0.937500 |
| Task 2 vs. Task 4 | F1 | CNN-LSTM | SVM | 0.062500 | 0.937500 |
| Task 2 vs. Task 4 | F1 | LSTM | LightGBM | 0.062500 | 0.937500 |
| Task 2 vs. Task 4 | F1 | LSTM | RF | 0.062500 | 0.937500 |
| Task 2 vs. Task 4 | F1 | LSTM | MLP | 0.062500 | 0.937500 |
| Task 2 vs. Task 4 | F1 | LSTM | SVM | 0.062500 | 0.937500 |
| Task 2 vs. Task 4 | F1 | LightGBM | RF | 0.465209 | 1 |
| Task 2 vs. Task 4 | F1 | LightGBM | MLP | 0.108809 | 0.937500 |
| Task 2 vs. Task 4 | F1 | LightGBM | SVM | 0.067889 | 0.937500 |
| Task 2 vs. Task 4 | F1 | RF | MLP | 0.285049 | 1 |
| Task 2 vs. Task 4 | F1 | RF | SVM | 0.437500 | 1 |
| Task 2 vs. Task 4 | F1 | MLP | SVM | 0.592980 | 1 |
|  |  |  |  |  |  |

Note: P_Uncorrected = raw p value from the Wilcoxon signed-rank test; P_Holm = Holm-adjusted p value for multiple comparisons.

Figure S1. SHAP-based feature importance in LR modeling under different conditions: (a) Task 1 vs. Task 2; (b) Task 3 vs. Task 4; (c) Task 1 vs. Task 3; (d) Task 2 vs. Task 4.
